# Baicalein inhibits inflammatory response and promotes osteogenic activity in periodontal ligament cells challenged with lipopolysaccharides

**DOI:** 10.1186/s12906-021-03213-5

**Published:** 2021-01-23

**Authors:** Manman Ren, Ya Zhao, Zhiqi He, Jian Lin, Chuchu Xu, Fen Liu, Rongdang Hu, Hui Deng, Yi Wang

**Affiliations:** 1 Department of Periodontics, School of Stomatology, Wenzhou Medical University, Wenzhou, Zhejiang China; 2 Department of Orthodontics, School of Stomatology, Wenzhou Medical University, Wenzhou, Zhejiang China; 3 Department of Histology and Embryology, Wenzhou Medical University, Wenzhou, Zhejiang China

**Keywords:** Periodontitis, Baicalein, Osteogenesis, Inflammation, MAPKs, Wnt/β-catenin

## Abstract

**Background:**

Periodontitis is a chronic infection initiated by oral bacterial and their virulence factors, yet the severity of periodontitis is largely determined by the dysregulated host immuno-inflammatory response. Baicalein is a flavonoid extracted from *Scutellaria baicalensis* with promising anti-inflammatory properties. This study aims to clarify the anti-inflammatory and osteogenic effects of baicalein in periodontal ligament cells (PDLCs) treated with lipopolysaccharides (LPS).

**Methods:**

Human PDLCs were incubated with baicalein (0–100 μM) for 2 h prior to LPS challenge for 24 h. MTT analysis was adopted to assess the cytoxicity of baicalein. The mRNA and protein expression of inflammatory and osteogenic markers were measured by real-time polymerase chain reaction (PCR), western blot and enzyme-linked immunosorbent assay (ELISA) as appropriate. Alkaline phosphatase (ALP) and Alizarin red S (ARS) staining were performed to evaluate the osteogenic differentiation of PDLCs. The expression of Wnt/β-catenin and mitogen-activated protein kinase (MAPK) signaling related proteins was assessed by western blot.

**Results:**

MTT results showed that baicalein up to 100 μM had no cytotoxicity on PDLCs. Baicalein significantly attenuated the inflammatory factors induced by LPS, including interleukin-1β (IL-1β), tumor necrosis factor-α (TNF-α), matrix metalloprotein-1 (MMP-1), MMP-2 and monocyte chemoattractant protein 1 (MCP-1) at both mRNA and protein level. Moreover, MAPK signaling (ERK, JNK and p38) was significantly inhibited by baicalein, which may account for the mitigated inflammatory response. Next, we found that baicalein effectively restored the osteogenic differentiation of LPS-treated PDLCs, as shown by the increased ALP and ARS staining. Accordingly, the protein and gene expression of osteogenic markers, namely runt-related transcription factor 2 (RUNX2), collagen-I, and osterix were markedly upregulated. Importantly, baicalein could function as the Wnt/β-catenin signaling activator, which may lead to the increased osteoblastic differentiation of PDLCs.

**Conclusions:**

With the limitation of the study, we provide in vitro evidence that baicalein ameliorates inflammatory response and restores osteogenesis in PDLCs challenged with LPS, indicating its potential use as the host response modulator for the management of periodontitis.

**Supplementary Information:**

The online version contains supplementary material available at 10.1186/s12906-021-03213-5.

## Background

Periodontitis, which affects about 5 to 15% of world populations, remain as a major global health burden. It is characterized by the accumulation of dental biofilm and resultant destruction of tooth-supporting tissues that eventually leads to multiple tooth loss [1, 2]. Moreover, periodontitis is known to be responsible for an increased risk of a series of systemic comorbidities, such as cardiovascular disease and diabetes mellitus [3, 4].

Periodontal pathogens, which mainly comprised of Gram-negative anaerobic bacterial, are required to initiate periodontitis. However, it is the dysregulated host immuno-inflammatory response that largely determines the extent and severity of periodontal disease [5]. The infection with the pathogenic microflora, together with their virulence factors like lipopolysaccharides (LPS) and gingipain, drives the influx of neutrophils and macrophages, and activates the constituent cells, e.g. periodontal ligament cells (PDLCs), releasing a plethora of inflammatory mediators that participates in the initiation and progression of periodontitis [5, 6]. Several signaling pathways have been implicated in the complex underlying molecular network of periodontitis. For example, mitogen-activated protein kinase (MAPK) and nuclear factor-kappaB (NF-κB) are known to regulate the expression of inflammatory mediators [7], and Wnt/β-catenin signaling is closely involved in bone turnover and remodeling [8].

Host modulation therapy with anti-inflammatory agents has now been used as a promising adjunct to conventional periodontal management which mainly deals with the bacterial challenge [9]. Baicalin, and its aglycone baicalein are the two major bioactive flavonoids extracted from *Scutellaria baicalensis* Georgi (Named Huang-qin in Chinese), which are widely used in traditional Chinese herbal medicine. It is well documented that baicalein and baicalin both have diverse functional activities such as osteogenesis, cardioprotective, anti-cancer and anti-diabetes, mainly due to their remarkable anti-inflammation and anti-oxidant efficacies [10–13].

In periodontal research, baicalin has been shown to significantly mitigate the ligature-induced alveolar bone breakdown in rats with experimental periodontitis [14], and this could be explained by its pro-osteogenic effect on PDLCs [15]. Yet, the underlying mechanism and relevant signaling pathways are poorly characterized. On the other hand, few reports have been focused on the modulatory effects of baicalein in periodontitis. A recent study suggested that baicalein induces the proliferation and formation of calcified nodules in PDLCs, while baicalin not [16]. Moreover, nano-encapsulated baicalein, rather than baicalin, exerts pronounced anti-inflammatory effect in gingival epithelial cells under inflammatory environment [17]. The diverse effects observed between baicalein and baicalin could be explained by their different structures and bioavailability. In addition, we have previously shown that baicalein enables the osteoblastic differentiation of PDLCs through Wnt/β-catenin signaling [18]. Therefore, this extended study aims to evaluate the effects of baicalein on the inflammatory and osteogenic factors in PDLCs challenged with LPS, as well as the underlying molecular mechanisms.

## Methods

### Cell culture and treatment

Human PDLCs were isolated and cultured according to our previously established protocol [19]. Cells were characterized by immunohistochemical analysis for vimentin and cytokeratin (1:200, BosterBio, China). Cells at Passage 3–4 were used in the following experiments. This study was approved by the Ethics Committee of School & Hospital of Stomatology, Wenzhou Medical University. Written Informed consents were obtained from all participants prior to the enrollment.

Baicalein (purity > 95%, Sigma, USA) was dissolved in dimethylsulfoxide (DMSO, Sigma, USA), and added to the culture medium to reach the indicated concentration (10–100 μM). DMSO without baicalein was used as control. 2 mL of PDLCs were firstly plated into 6-well plates at a density of 2 × 10^5^ cells/well. After confluence, cells were incubated with baicalein for 2 h prior to LPS (200 ng/ml, *Escherichia coli* O55:B5, L2880, Sigma, USA) stimulation for 24 h, were harvested for following analytical experiments, except that the phosphorylation of MAPK family proteins were assessed after 1 h of LPS treatment. The concentration of LPS was applied at 200 ng/ml as it showed pronounced pro-inflammatory effects within the least concentration based on our preliminary results. To inhibit or activate the specific signaling pathway, cells were treated with MAPK inhibitors (20 μM, PD98059, ERK inhibitor; SP600125, JNK inhibitor; SB203580, p38 inhibitor, Calbiochem, USA) or Wnt3a (100 ng/ml, Wnt/β-catenin activator, Sigma, USA) for 24 h together with LPS.

### Cell viability assay

The effects of baicalein on the proliferation of PDLCs was assessed by a 3-(4,5-dimethylthiazol-2-yl)-2,5-diphenyltetrazolium bromide (MTT) analysis. Cells were plated into 96-well plates (2 × 10^3^ cells/well) overnight and incubated with increasing concentrations of baicalein (0, 10, 20, 40, 60, 80, 100 μM) for 12, 24, 36, 72 and 96 h respectively. 20 μL of MTT solution (5 mg/ml, Sigma, USA) was added to the wells and kept in dark 4 h prior to the indicated time. The formazan crystals were then dissolved in 150 μL of DMSO and the absorbance was measured at 570 nm by a microplate reader (Infinite M200 PRO, Tecan, China).

### Alkaline phosphate (ALP) and alizarin red S (ARS) staining

Following the indicated treatment, cells were cultured in the osteogenic medium containing 10^− 5^ mM dexamethasone, 50 μg/mL ascorbic acid, and 5 mM β-glycerophosphate for 7 days. ALP staining was subsequently performed with the use of the BCIP/NBT Alkaline Phosphatase Color Development kit as per the manufacturer’s protocol (Beyotime, China). The ARS staining was conducted following 21 days of PDLCs culture in the osteogenic medium. Cells were fixed and stained with l mg/ml of Alizarin Red solution (Sigma, USA). The red mineralized nodules were observed under phase contrast microscope.

### Enzyme-linked immunosorbent assay (ELISA)

After the indicated treatment, the culture supernatants were collected and stored under − 70 °C until analysis. ELISA kits (Multisciences, China) was used to quantify the concentrations of interlukein-1β (IL-1β), tumor necrosis factor-α (TNF-α), matrix metalloprotein-1 (MMP-1), MMP-2 and monocyte chemoattractant protein 1 (MCP-1) in the culture supernatants according to the manufacturer’s protocol.

### RNA preparation and real-time quantitative polymerase chain reaction (RT-qPCR)

Following the indicated treatment, total RNA was extracted by Trizol reagent (Invitrogen, USA). The concentration and purity of RNA was determined by spectrophotometer. An equal mass of RNA (1 μg) was reverse-transcribed into cDNA with PrimeScript™ RT Reagent Kit (Takara, Japan) following the manufacturer’s protocol. RT-qPCR was performed using LightCycler 480 SYBR Green I Master (Roche, USA) on a LightCycler 480 system (Roche, USA) under the following settings: 95 °C at 30 s for activation, 40 cycles of 95 °C for 5 s, 60 °C at 20 s for annealing, and extension at 60 °C for 1 min. The primers was listed in Table 1. The relative mRNA expression was calculated using the 2^-ΔΔCt^ method [20].

**Table 1:** Primer Sequences

Gene	Forward (5′-3′)	Reverse (5′-3′)
RUNX2	CCCGTGGCCTTCAAGGT	CGTTACCCGCCATGACAGTA
OSX	ACCTACCCATCTGACTTTGCTC	CCACTATTTCCCACTGCCTTG
COL-I	CCAGAAGAACTGGTACATCAGCAA	CGCCATACTCGAACTGGAATC
IL-1β	CGTTACCCGCCATGACAGTA	TGCTGTAGTGGTGGTCGGAGA
TNF-α	CCTGGTATGAGCCCATCTATC	GGTTGGATGTTCGTCCTCCTC
MMP-1	TCGTGGTTCCAACTCGGTTT	GCGGCCCTCGAAGATGA
MMP-2	CCGTCGCCCATCATCAA	AGATATTGCACTGCCAACTCT
MCP-1	AGCATGACAAGGCCTGCGTC	TGGCACCCAGCACAATGAA
β-actin	TGGCACCCAGCACAATGAA	CTAAGTCATAGTCCGCCTAGAAGCA

### Protein preparation and Western blot

After the indicated treatment, cells were lysed in ice-cold RIPA buffer (Fdbio, China). Following centrifugation, the supernatant was collected and quantitatively determined with a BCA protein assay kit (Beyotime, China). 20 μg of protein was separated by 10% SDS-polyacrylamide gel electrophoresis (PAGE) and transferred to polyvinylidene difluoride membrane. The membranes were then incubated with the following primary antibody at 4 °C overnight: Runt-related transcription factor 2 (RUNX2, 1:1000, No. 8486, CST, USA), β-catenin (1:1000, No. 8480, CST, USA), LEF1 (1:1000, No. 2230, CST, USA), Cyclin D (1:1000, No. 2978, CST, USA), β-actin (1:1000, No. 4970, CST, USA), Collagen-I (COL-I, 1:1000, No. sc-8785, Santa Cruz, USA), Osterix (OSX, 1:2000, No. ab94744, Abcam, USA), ERK (1:1000, No. 4695, CST, USA), phospho-ERK (p-ERK, 1:1000, No. 4370, CST, USA), p38 (1:1000, No. 8690, CST, USA), phospho-p38 (p-p38, 1:1000, No. 4511, CST, USA), JNK (1:1000, No. 9252, CST, USA), phospho-JNK (p-JNK, 1:1000, No. 4668, CST, USA). Following the incubation with horseradish peroxidase (HRP)-conjugated secondary antibodies for 2 h, the protein bands were visualized by Bio-Rad ChemiDocTM XRS+ with ECL enhanced chemiluminescence kit. The intensity of the bands was quantified by ImageJ software (Version 1.52, JAVA, Win, NIH).

### Statistics

All the experiments were performed in at least triplicate independently and the results were presented as mean ± standard deviation (SD). The comparison was made by one-way analysis of variance (ANOVA) with post hoc LSD test. *P* value < 0.05 indicates statistical significance. All statistical analysis was performed on SPSS 19.0 (IBM, USA).

## Results

### Characterization of PDLCs

**Fig. 1:**
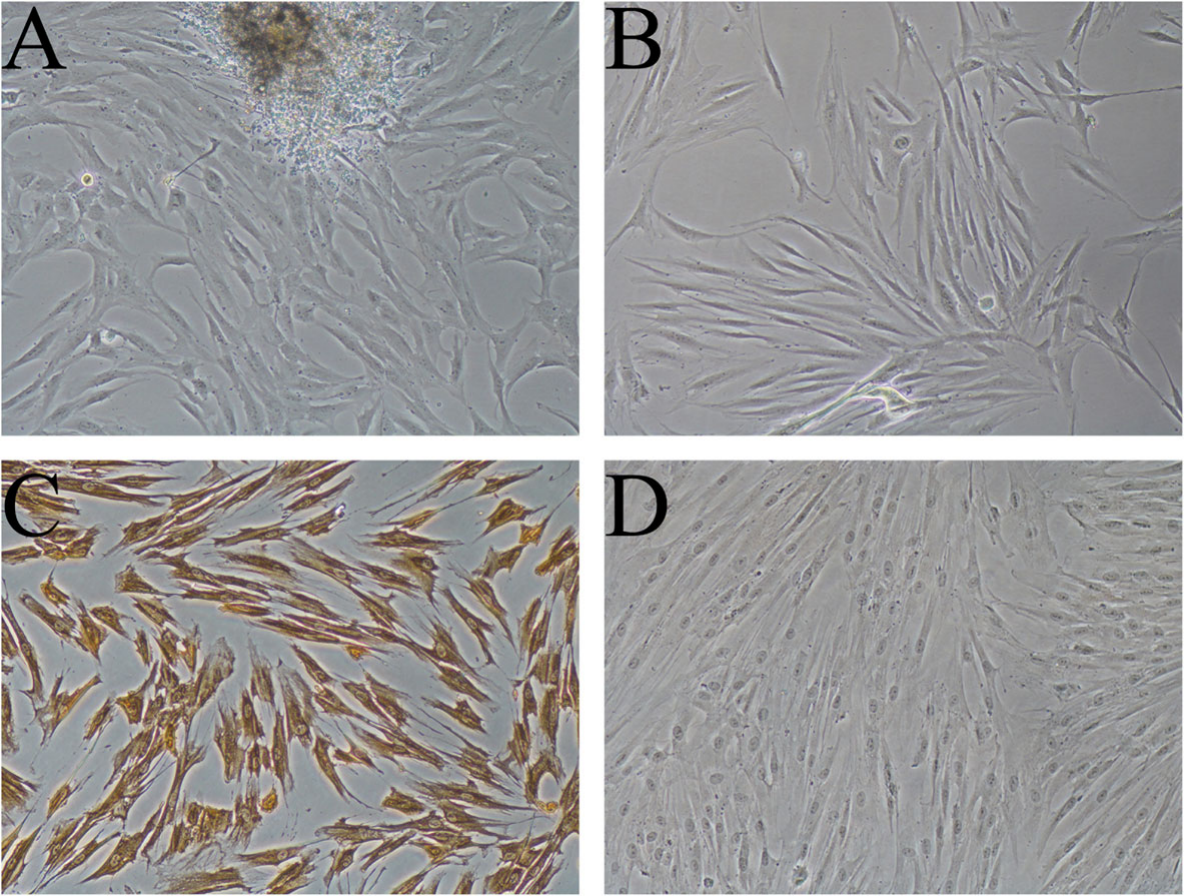
The characterization of PDLCs. Primary culture of PDLCs at the Day 7 (×100) (**a**); Morphology of PDLCs at Passage 1 (× 100) (**b**); Immunohistochemistry staining results of PDLCs, positive for vimentin (× 100) (**c**) and negative for cytokeratin (× 100) (**d**)

On day 5–7, PDLCs growing out of the tissues were observed under microscope (Fig. 1a). After passaging, cells displayed a spindle-like shape, which resembled the typical morphology of fibroblast cells (Fig. 1b). The immunohistochemical staining was positive for vimentin (Fig. 1c) and negative for cytokeratin (Fig. 1d). Collectively, these results suggest that the cells could be identified as PDLCs, which was consistent with previous findings [21, 22].

### Baicalein did not affect cell viability in PDLCs

**Fig. 2:**
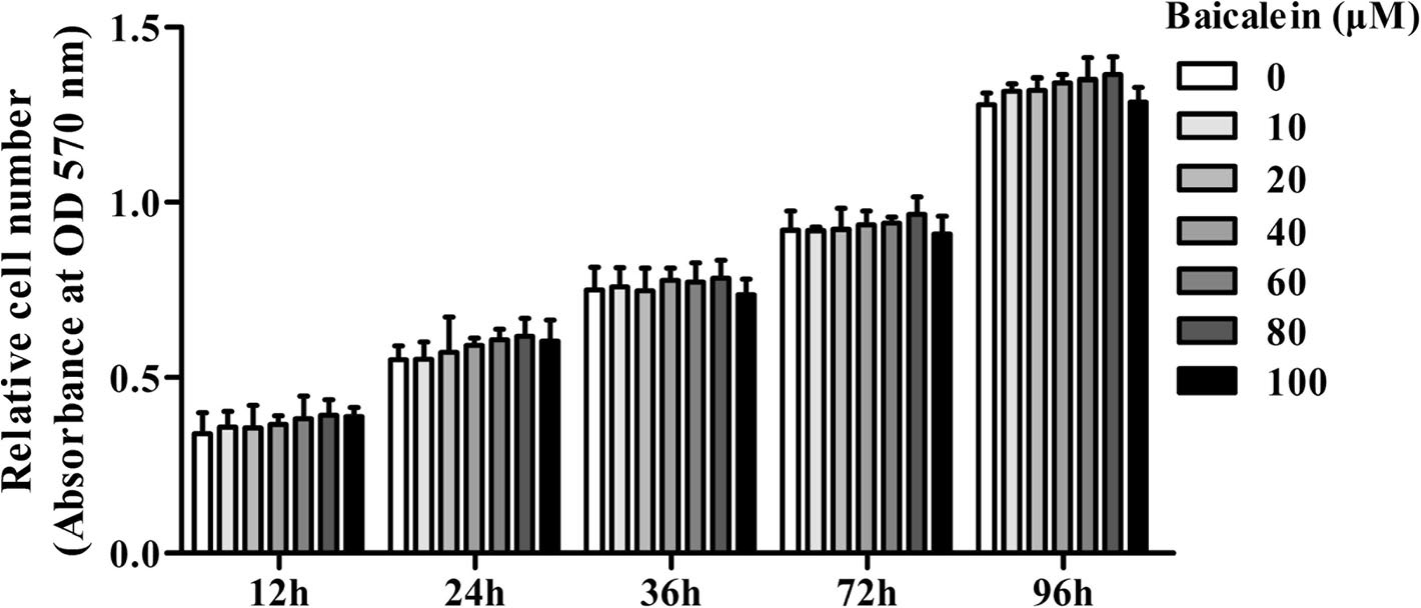
The effects of baicalein on the viability of PDLCs determined by the MTT assay. It shows that baicalein (20, 40, or 80 μM) did not affect the cell viability up to 96 h

As shown in Fig. 2, MTT assay showed that baicalein at concentrations ranging from 10 to 100 μM did not affect cell viability up to 96 h, indicating a good biosafety.

### Baicalein suppressed inflammation and MAPK signaling in LPS-treated PDLCs

**Fig. 3:**
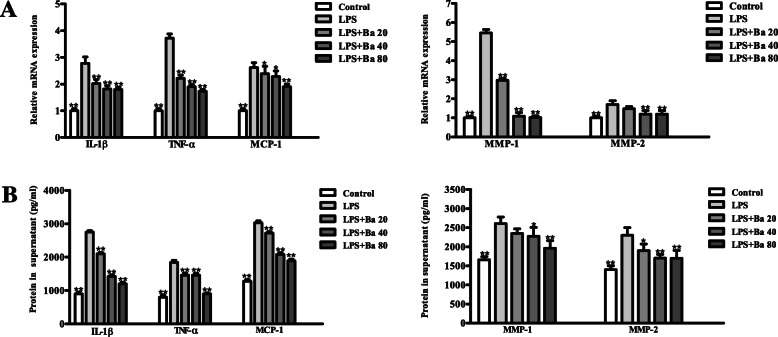
Baicalein antagonizes the LPS-induced inflammatory factors in PDLCs. Cells were pretreated with baicalein (20, 40, or 80 μM) for 2 h prior to incubation with LPS for 24 h. The mRNA expressions were analyzed by qRT-PCR (**a**) and the protein concentrations in the supernatant were evaluated by ELISA (**b**). The results showed that 40 and 80 μM of baicalein significantly reduced the mRNA and cytokine concentrations of IL-1β, TNF-α, MCP-1, MMP-1 and MMP-2. *p < 0.05, **p < 0.01, statistical significant difference in comparison to cells treated with LPS alone

**Fig. 4:**
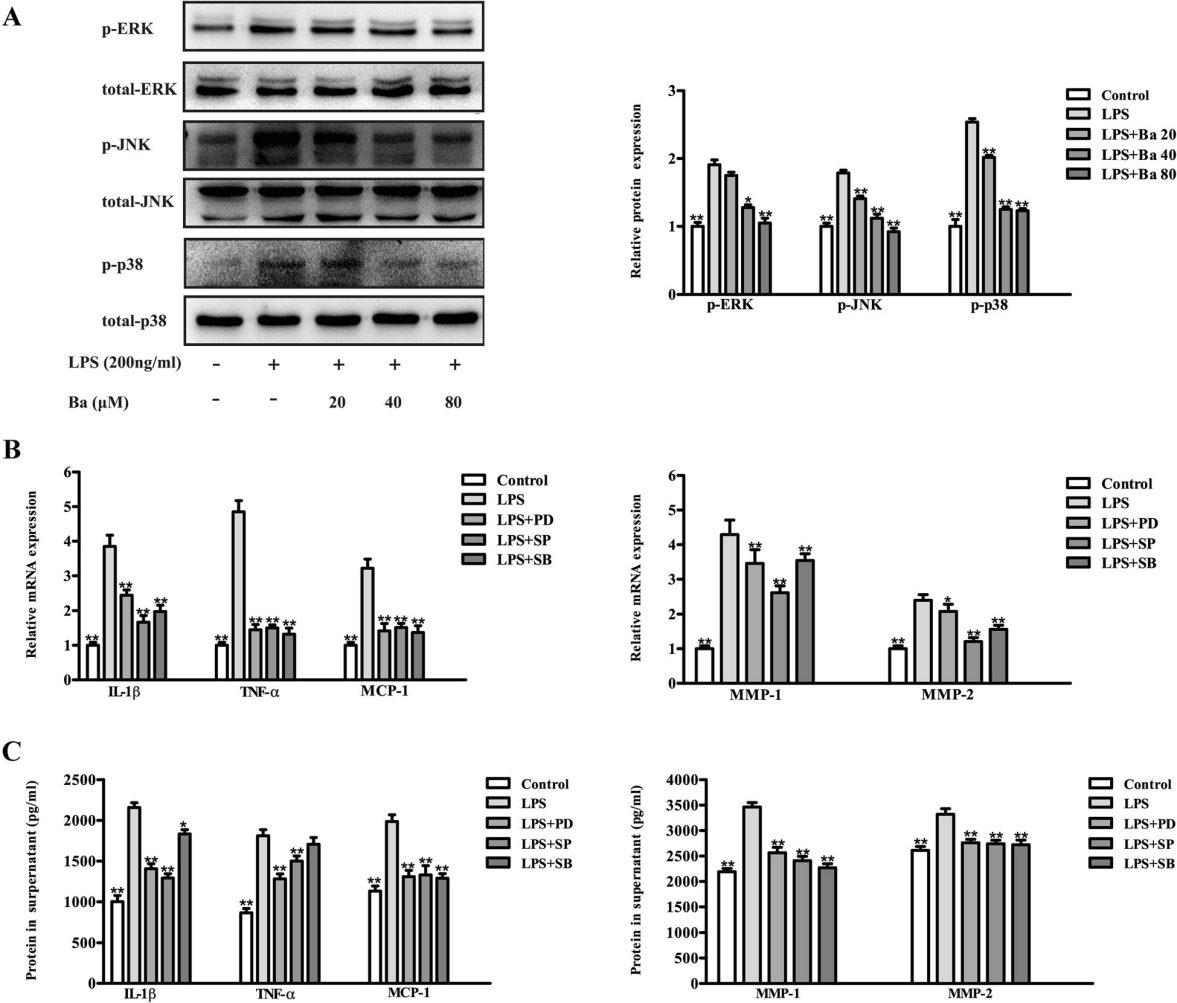
Baicalein acts as the MAPK signaling inhibitor to down-regulate the expressions of inflammatory mediators of PDLCs challenged with LPS. The western blot results showed that LPS induced the phosphorylation of MAPK family proteins (ERK, JNK and p38) could be inhibited by baicalein (**a**). The inhibition of MAPK signaling (PD98059, PD, ERK inhibitor; SP600125, SP, JNK inhibitor; SB203580, SB, p38 inhibitor) significantly reduced the mRNA expressions (**b**) and protein concentrations (**c**) of IL-1β, TNF-α, MCP-1, MMP-1 and MMP-2. *p < 0.05, **p < 0.01, statistical significant difference in comparison to cells treated with LPS alone

As indicated in Fig. 3a-d, 20, 40 and 80 μM of baicalein all effectively downregulated the LPS-induced transcripts and supernatant concentrations of inflammatory cytokines including IL-1β, TNF-α and MCP-1 (*P* < 0.05). While the mRNA and protein expressions of MMP-1 and MMP-2 were found to be significantly decreased by the pre-treatment of baicalein at the concentrations of 40 and 80 μM (*P* < 0.05). The immunoblotting results showed that treatment with LPS for 1 h markedly induced the phosphorylation of the MAPK family proteins, namely ERK, JNK and p38 (*P* < 0.05, Fig. 4a). Moreover, the mRNA and protein expressions of inflammatory factors (IL-1β, MCP-1, MMP-1 and -2) up-regulated by LPS could be significantly attenuated by the selective inhibitor of ERK, JNK and p38 respectively (*P* < 0.05). The transcripts and protein level of TNF-α were reduced via the inhibition of ERK and JNK signaling only (Fig. 4b & c). Importantly, baicalein impeded the phosphorylation of ERK, JNK and p38 induced by LPS (Fig. 4a). Given the effect of MAPK inhibitors on the suppression of inflammation, it is suggested that baicalein may mitigate the expressions of inflammatory factors in LPS-treated PDLCs through MAPK signaling inhibition. The uncropped blots for Fig. 4a can be viewed in Fig. S1 in the online supplementary materials.

### Baicalein promotes osteogenesis and Wnt/β-catenin signaling in LPS-treated PDLCs

**Fig. 5:**
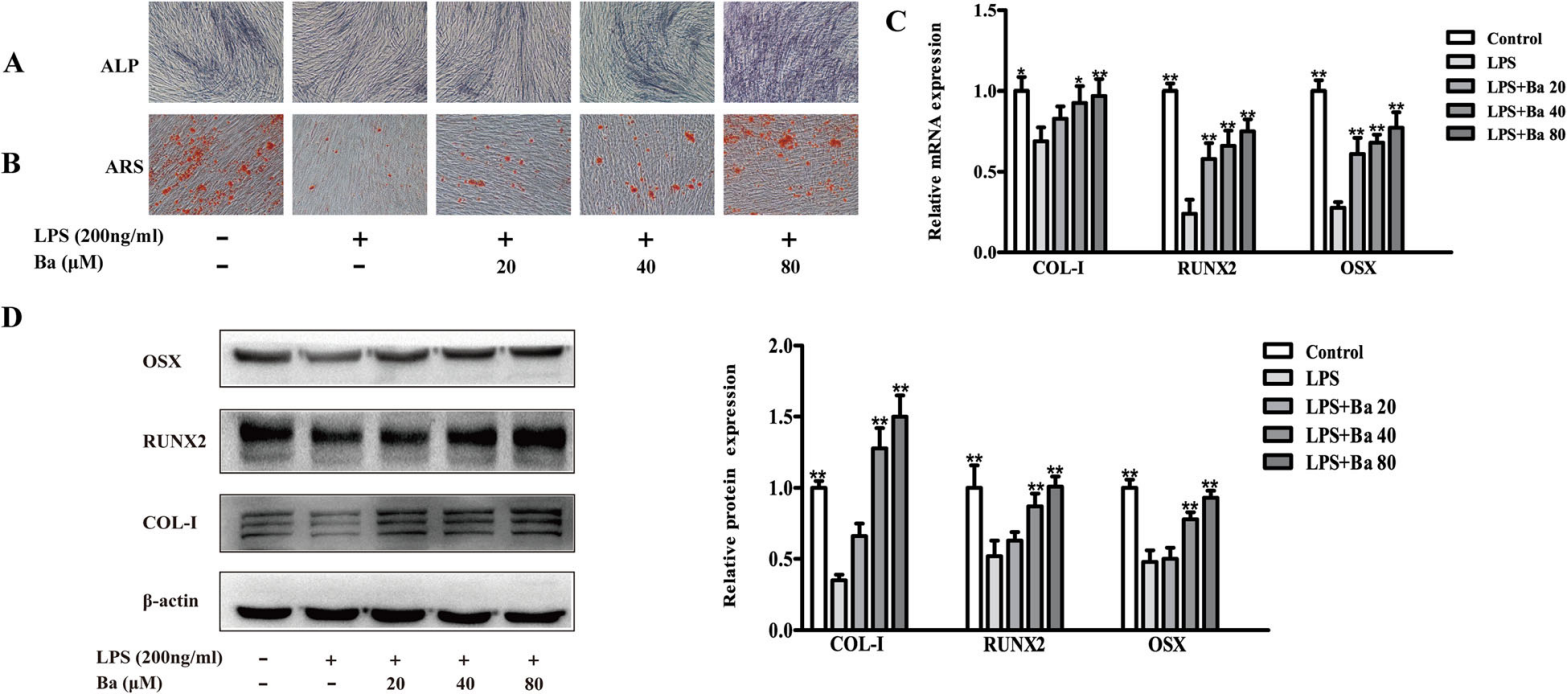
Baicalein restores the osteogenesis of PDLCs challenged with LPS. Cells were pre-treated with baicalein (20, 40, or 80 μM) for 2 h prior to incubation with or without LPS for 24 h. Baicalein effectively increased the ALP (**a**) and ARS (**b**) staining of LPS-treated PDLCs. The mRNA (**c**) and protein (**d**) expressions of COL-1, RUNX2 and OSX were dose-dependently up-regulated by baicalein. *p < 0.05, **p < 0.01, statistical significant difference in comparison to cells treated with LPS alone.

The early and late osteogenic differentiation of PDLCs was assessed by ALP and ARS assay respectively. It was shown that the baicalein markedly increased the intensity of ALP staining and the number of red mineralized nodules in LPS-treated cells (Fig. 5a & b). Accordingly, the mRNA and protein expressions of osteogenic markers including COL-I, RUNX2 and OSX, which were downregulated by LPS, were significantly recovered by baicalein at 40 and 80 μM (*P* < 0.01, Fig. 5c & d). The uncropped blots for Fig. 5d were shown in Fig. S2 in the online supplementary materials.

**Fig. 6:**
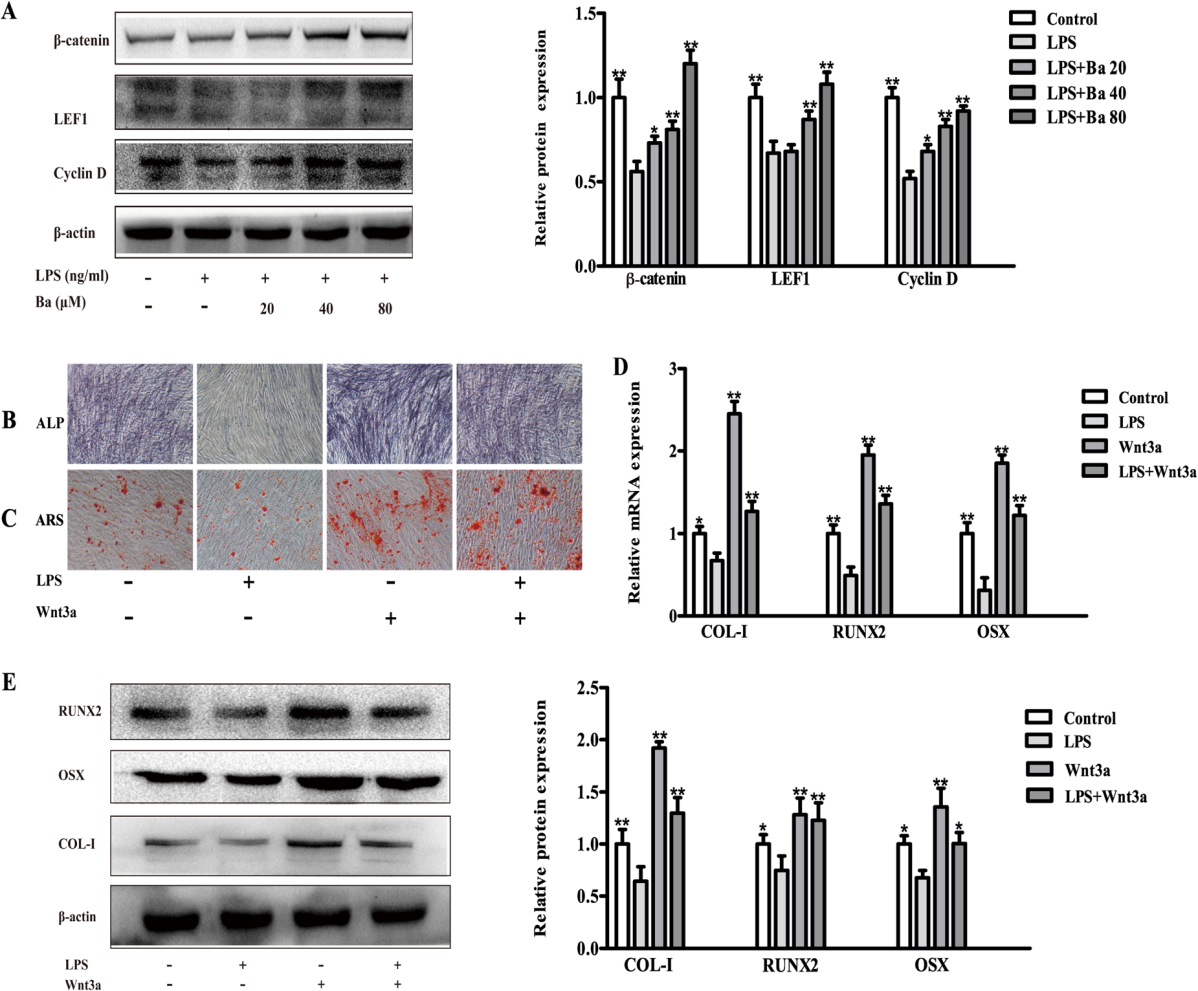
Baicalein acts as the Wnt/β-catenin signaling activator to up-regulate the osteogenesis of PDLCs challenged with LPS. The LPS-decreased expression of Wnt/β-catenin signaling related protein was recovered by baicalein (**a**). The ALP staining (**b**) and mineralized nodule formation (**c**) inhibited by LPS, was found to be restored by Wnt/β-catenin signaling activator, wnt3a. The mRNA (**d**) and protein (**e**) expressions of COL-1, RUNX2 and OSX, which were down-regulated by LPS, were significantly increased following the treatment of baicalein. *p < 0.05, **p < 0.01, statistical significant difference in comparison to cells treated with LPS

The modulatory effect of Wnt/β-catenin signaling in LPS-treated PDLCs was further investigated. We found that LPS reduced the expression of Wnt/β-catenin signaling related proteins including β-catenin, LEF1 and Cyclin D (Fig. 6a). Furthermore, the activation of Wnt/β-catenin signaling by Wnt3a significantly increased the osteogenic differentiation of PDLCs treated with LPS (Fig. 6b & c), accompanied by the elevated mRNA and protein expressions of osteogenic markers (COL-I, RUNX2 and OSX, *P* < 0.05, Fig. 6d & e). Provided that baicalein might act as a Wnt/β-catenin signaling activator to induce the protein expressions of β-catenin, LEF1 and Cyclin D (Fig. 6a), we assumed that baicalein could recover the osteogenic differentiation of LPS-treated PDLCs via the activation of Wnt/β-catenin signaling. The uncropped blots for Fig. 6a and e were presented in Fig. S3 and 4 respectively in the online supplementary materials.

## Discussion

Periodontitis is essentially the consequence of the dysregulated host-microbial interactions. Recently, therapeutic management towards the host immuno-inflammatory response has been proposed as a promising approach [23]. Indeed, several compounds from Chinese medicinal herbs have been suggested for the treatment of periodontal disease, e.g., osthole from *Cnidium monnieri* [24], shikonin from *Radix Arnebiae* [25], psoralen and angelicin from *Psoraleae* [26]. The present study demonstrated that baicalein increased osteogenic differentiation and decreased inflammation in LPS-treated PDLCs, suggesting that baicalein could be potentially used in addition to dental biofilm management.

PDLCs are closely involved in repair, remodeling and regeneration of the adjacent cementum and bone, contributing to the maintenance of tooth supporting tissues. It is demonstrated that PDLCs exhibit a decreased osteogenicity in the presence of inflammatory microenvironment, resulting in the breakdown of the adjacent tissues [27]. *E. coli*-LPS is a well-known potent inducer of inflammatory cytokines and chemokines, and therefore used to mimic microenvironment in the present study. Our study showed that LPS inhibited the osteogenic differentiation of PDLCS, together with a markedly increased secretion of inflammatory mediators, confirming the results of previous studies [28, 29].

The inflammatory cytokines play a crucial role in the host response to periodontal infection. Clinical evidence suggested that the gingival crevicular levels of IL-1β and TNF-α are positively associated with the onset and progression of periodontitis [30]. In addition, an increased level MCP-1 enables the immune cells to infiltrate and accumulate into the gingival sulcus, producing a plethora of pro-inflammatory cytokines. These cytokines may in turn amplify the chemotactic signals that generated by PDLCs challenged with inflammatory agents [5]. Moreover, MMP-1 and -2 are important enzymes for matrix degradation. Their upregulation are responsible for the turnover of periodontal ligament collagens and subsequent attachment loss [31]. The anti-inflammatory property of baicalein has been validated in immune cells, endothelial cells and oral epithelial cells [17, 32, 33], whether such effect could be extended to PDLCs remains to be clarified. To the best of our knowledge, the present study for the first time, showed that baicalein effectively reduced these inflammatory mediators (IL-1β, TNF-α, MCP-1, MMP-1 & -2) induced by LPS in PDLCs.

MAPK signaling has been widely reported to be the potential target for inflammatory stimuli [34]. Indeed, pro-inflammatory agents have been demonstrated to induce the phosphorylation of MAPK family proteins including ERK1/2, JNK and p38 in PDLCs [35, 36]. Accordingly, we found that the inhibition of ERK1/2, JNK and p38 pathways resulted in an reduced expression of inflammatory cytokines. Importantly, baicalein impeded the MAPK signaling activated by LPS, potentially suggesting that the anti-inflammatory effect was mediated by MAPK signaling. Moreover, the suppression of MAPK signaling by baicalein has been demonstrated to be regulated by the upstream Toll-like Receptor-4 (TLR-4) [37, 38]. Since TLR2/4 are highly functional on PDLCs exposed to gram-negative bacterial challenge [39], future studies investigating the TLR-MAPK axis are highly warranted.

The osteogenic effects of baicalein is controversial. It is reported that baicalein slightly suppressed the ALP activity of MC3TE-1 cells [40]. While another group consistently demonstrated that baicalein promotes the osteoblastic differentiation in MC3TE-1, and inhibits the bone resorption by inducing the apoptosis of mature osteoclasts [41, 42]. Moreover, since the regeneration capacity of inflamed PDLCs is impaired, it is important to investigate the osteogenic effect of baicalein under inflammatory microenvironment [43]. ALP staining (indicator of early phase osteoblastic differentiation) and mineralized nodules (indicator of late phase osteoblastic differentiation) were found to be markedly increased by baicalein in LPS-challenged cells in our study. RUNX-2, is a master regulator of osteoblastic differentiation, who serves as the upstream activator of OSX and COL-1. Osterix is actively involved in the maturity and terminal differentiation of osteoblast, and collagen-I is a bone protein secreted exclusively by osteoblasts [44, 45]. Our result showed that baicalein dose-dependently up-regulated the transcripts and protein expressions of RUNX-2, OSX and COL-1 in LPS-challenged cells, which were in accordance with ALP and ARS findings.

It is well acknowledged that activation of Wnt/β-catenin promotes osteoblastic differentiation, while the disruption of which leads to impaired osteogenesis [46]. Besides, RUNX2, which was found to be elevated by baicalein, is a direct target of canonical Wnt signaling [47]. We therefore investigated the effects of baicalein on Wnt/β-catenin signaling. In the present study, osteogenic capacity reduced by LPS could be almost completely reversed by the addition of Wnt3a, a potent activator of Wnt/β-catenin signaling. Notably, Wnt targeted proteins (LEF1, Cyclin D and β-catenin) were significantly induced by baicalein, suggesting that baicalein may function as Wnt/β-catenin signaling activator, promoting the osteogenicity of PDLCs under inflammatory microenvironment. This was in accordance with previous studies in which agents that activating Wnt/β-catenin signaling could stimulate PDLCs osteoblastic differentiation in the inflammatory environment [48, 49].

The present study indicated that 2 h of pre-treatment with baicalein effectively attenuated the LPS-induced inflammatory factors, as well as the LPS-inhibited osteogenic differentiation in PDLCs. Whereas it may take 24 to 48 h of co-incubation with baicalin to exert its protective effect on PDLCs, as indicated by previous studies [50, 51]. Given the low half-life and rapid elimination from the local sites in the oral cavity for baicalein and baicalin [17], a short period of time (2 h) may better mimic the in vivo conditions. Nevertheless, future studies are highly warranted to verify the in vivo effects and relevant molecular mechanisms of baicalein in the treatment of periodontitis. Moreover, the in vivo efficacy and biosafety of baicalein, in comparison to baicalin and other chemical anti-inflammation agents, should be investigated to determine the potential candidate for clinical use.

Several limitations have to be addressed. LPS derived from *E. coli* was adopted to create inflammatory environment in our study. Although LPS originated from *E. coli* and the major periodontal pathogen *P. gingivalis*, show somewhat different molecular structure, both of them could induce the production of inflammatory cytokines in PDLCs, such as IL-6, IL-8 and MCP-1 [52, 53]. Indeed, *E. coli* derive LPS have been applied to oral cells in a plenty of studies to represent the inflammatory state of periodontal disease [54–56]. The osteogenesis of PDLCs in inflammatory environment is complex, involving an array of transcription factors, signaling pathways. This study only investigated a subset of pathways involved in the inflammation and osteogenesis. Future studies are needed to investigate the comprehensive signaling network in an attempt to better elucidate the underlying mechanisms of baicalein.

## Conclusions

In conclusion, the present study demonstrated that baicalein antagonizes inflammation together with the inhibition of MAPK signaling, and induces osteogenicity and Wnt/β-catenin signaling in PDLCs challenged with LPS. These results revealed the potential therapeutic implication of baicalein as the host response modulator for the treatment of periodontitis.

## Supplementary Information

**Additional file 1.**

## Data Availability

The datasets used and/or analyzed during the current study are available from the corresponding author on reasonable request.

## Abbreviations

PDLCs: periodontal ligament cells;
LPS: lipopolysaccharides
PCR: polymerase chain reaction
ELISA: enzyme-linked immunosorbent assay
ALP: alkaline phosphatase
ARS: alizarin red S
IL: interleukin
TNF-α: tumor necrosis factor-α
MMP: matrix metalloprotein
MCP: monocyte chemoattractant protein
RUNX2: runt-related transcription factor 2
MAPK: mitogen-activated protein kinase
NF-κB: nuclear factor-kappaB
DMSO: dimethylsulfoxide
PAGE: polyacrylamide gel electrophoresis
HRP: horseradish peroxidase
ANOVA: one-way analysis of variance
SD: standard deviation
TLR: toll-like Receptor
