# Supplementary material for: Baicalein inhibits inflammatory response and promotes osteogenic activity in periodontal ligament cells challenged with lipopolysaccharides
Source: BMC Complement Med Ther. 2021 Jan 23;21:43. doi: 10.1186/s12906-021-03213-5 (PMC7824944; doi:10.1186/s12906-021-03213-5)
Supplement: Supplementary file 1 — Additional file 1. [file 12906_2021_3213_MOESM1_ESM.docx]

**Baicalein inhibits inflammatory response and promotes osteogenic activity in periodontal ligament cells challenged with lipopolysaccharides**

Manman Ren^1^, Ya Zhao^1^, Zhiqi He^1^, Jian Lin^1^, Chuchu Xu^1^, Fen Liu^3^, Rongdang Hu^2^, Hui Deng^1^, Yi Wang^2^

^1^Department of Periodontics, School of Stomatology, Wenzhou Medical University, Wenzhou, Zhejiang, China

^2^Department of Orthodontics, School of Stomatology, Wenzhou Medical University, Wenzhou, Zhejiang, China

^3^Department of Histology and Embryology, Wenzhou Medical University, Wenzhou, Zhejiang, China

**Supplementary results**


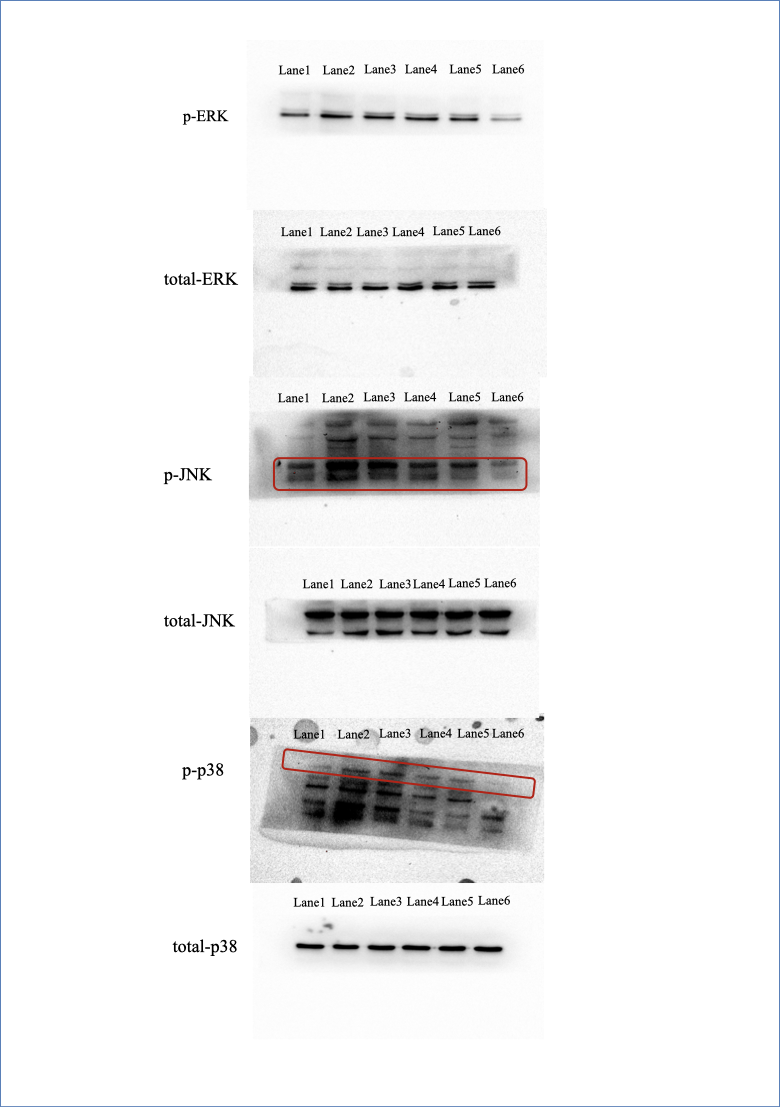


Fig. S1. Uncropped blots for Fig. 4A. Lane 1, Control group; Lane 2, LPS; Lane 3, LPS + Baicalein 20 𝜇M; Lane 4, LPS + Baicalein 40 𝜇M; Lane 5, LPS + Baicalein 80 𝜇M group; Lane 6: Baicalein 80 𝜇M. The red box indicates the specified band (based on the molecular weight) included in the final analysis. Lane 6 was not include in the final results according to the study design and grouping.


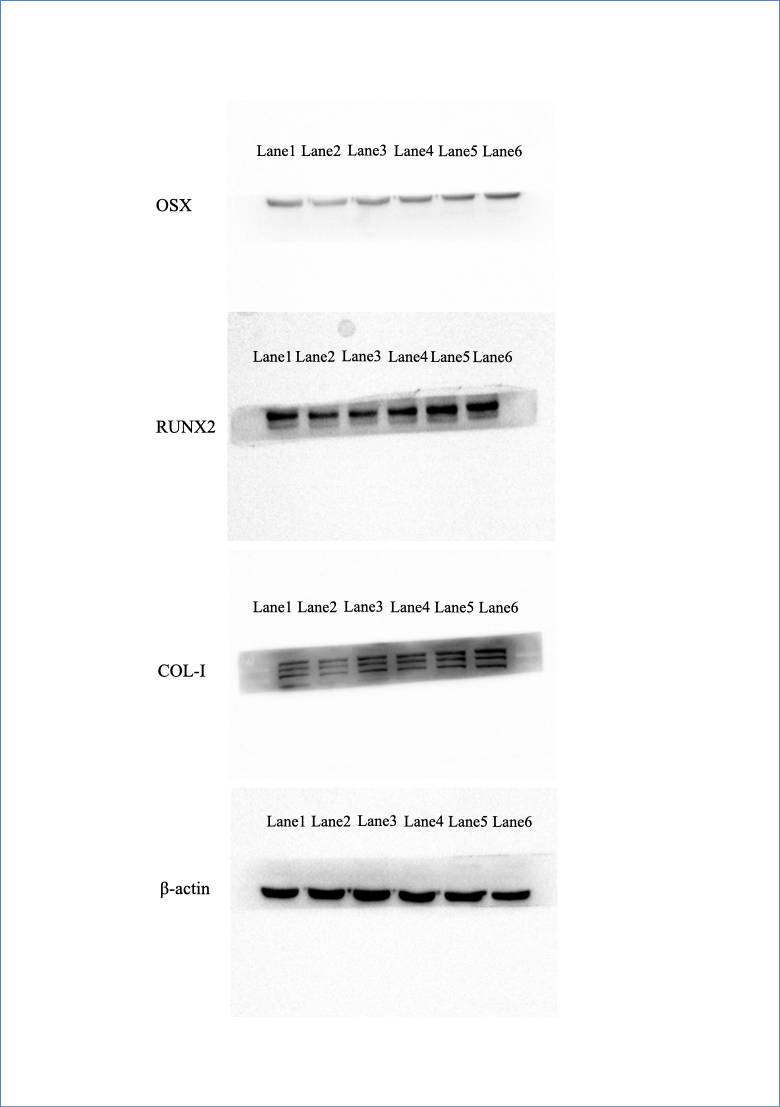


Fig. S2. Uncropped blots for Fig. 5D. Lane 1, Control group; Lane 2, LPS; Lane 3, LPS + Baicalein 20 𝜇M; Lane 4, LPS + Baicalein 40 𝜇M; Lane 5, LPS + Baicalein 80 𝜇M group; Lane 6: Baicalein 80 𝜇M. Lane 6 was not include in the final results according to the study design and grouping.


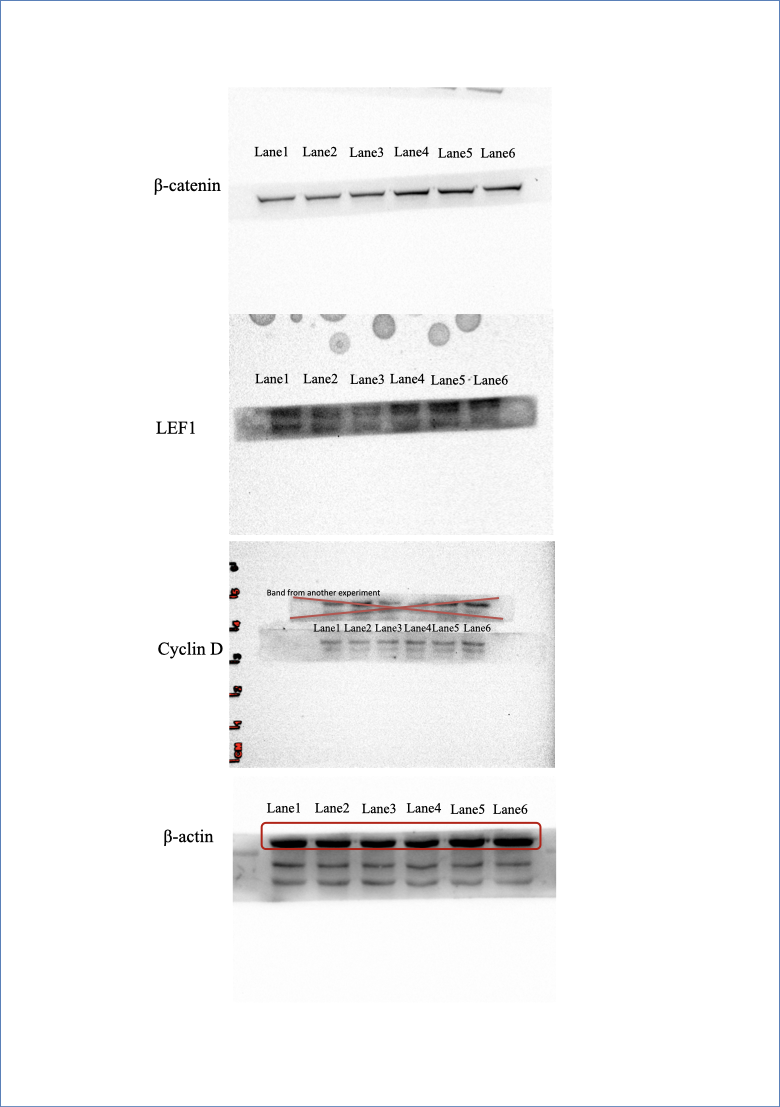


Fig. S3. Uncropped blots for Fig. 6A. Lane 1, Control group; Lane 2, LPS; Lane 3, LPS + Baicalein 20 𝜇M; Lane 4, LPS + Baicalein 40 𝜇M; Lane 5, LPS + Baicalein 80 𝜇M group; Lane 6: Baicalein 80 𝜇M. The red box indicates the specified band (based on the molecular weight) included in the final analysis. Lane 6 was not include in the final results according to the study design and grouping.


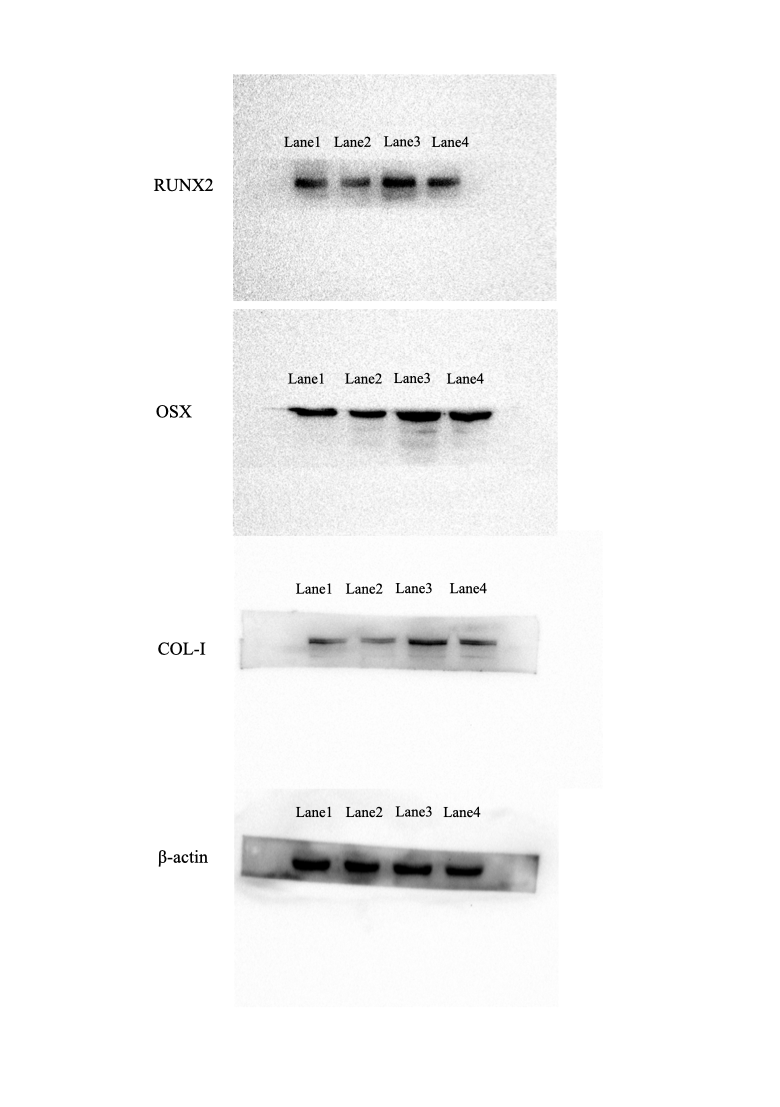


Fig. S4. Uncropped blots for Fig. 6E. Lane 1, control group; Lane 2, LPS; Lane 3, Wnt 3a; Lane 4, LPS+ Wnt 3a.
